# Spatial Distribution and Receptor Specificity of Zebrafish Kit System - Evidence for a Kit-Mediated Bi-Directional Communication System in the Preovulatory Ovarian Follicle

**DOI:** 10.1371/journal.pone.0056192

**Published:** 2013-02-08

**Authors:** Kai Yao, Wei Ge

**Affiliations:** School of Life Sciences and Centre for Cell and Developmental Biology, The Chinese University of Hong Kong, Shatin, New Territories, Hong Kong, China; Universitat de Barcelona, Spain

## Abstract

Consisting of Kit ligand and receptor Kit, the Kit system is involved in regulating many ovarian functions such as follicle activation, granulosa cell proliferation, and oocyte growth and maturation. In mammals, Kit ligand is derived from the granulosa cells and Kit receptor is expressed in the oocyte and theca cells. In the zebrafish, the Kit system contains two ligands (Kitlga and Kitlgb) and two receptors (Kita and Kitb). Interestingly, Kitlga and Kitb are localized in the somatic follicle cells, but Kitlgb and Kita are expressed in the oocyte. Using recombinant zebrafish Kitlga and Kitlgb, we demonstrated that Kitlga preferentially activated Kita whereas Kitlgb specifically activated Kitb by Western analysis for receptor phosphorylation. In support of this, Kitlgb triggered a stronger and longer MAPK phosphorylation in follicle cells than Kitlga, whereas Kitlga but not Kitlgb activated MAPK in the denuded oocytes, in agreement with the distribution of Kita and Kitb in the follicle and their specificity for Kitlga and Kitlgb. Further analysis of the interaction between Kit ligands and receptors by homology modeling showed that Kitlga-Kita and Kitlgb-Kitb both have more stable electrostatic interaction than Kitlgb-Kita or Kitlga-Kitb. A functional study of Kit involvement in final oocyte maturation showed that Kitlga and Kitlgb both suppressed the spontaneous maturation significantly; in contrast, Kitlgb but not Kitlga significantly promoted 17α, 20β-dihydroxy-4-pregnen-3-one (DHP) -induced oocyte maturation. Our results provided strong evidence for a Kit-mediated bi-directional communication system in the zebrafish ovarian follicle, which could be part of the complex interplay between the oocyte and the follicle cells in the development of follicles.

## Introduction

Kit ligand, also referred to as stem cell factor (SCF), mast cell growth factor (MGF) or steel factor (SF), is the product of the *Steel* (*Sl*) locus in mice [1]–[3] and its receptor, Kit, is encoded by the *White Spotting* (*W*) locus [2]–[7]. In the mouse ovary, Kit ligand is derived from the granulosa cells while its receptor Kit is restricted in the oocytes and theca cells [8], [9], suggesting a Kit ligand-mediated paracrine signaling from the granulosa cells to the oocytes as well as the theca cells.

It has been documented in mammals that Kit ligand is involved in promoting growth and survival of oocytes [10]–[13] but maintaining the arrest of meiosis [14], [15] by direct activation of Kit on the oocytes. These effects represent part of the mechanisms by which the follicle cells support the development of oocytes, which has long been considered an example of granulosa cell-to-oocyte communication in the ovarian follicle. Interestingly, Kit ligand also has mitogenic effect on the granulosa cells despite the lack of its receptor Kit in these cells; however, this effect requires the existence of oocytes [12], [16]. It has been postulated that Kit ligand may exert its mitogenic effect on the granulosa cells indirectly by inducing an oocyte-derived factor that in turn acts on the granulosa cells [17].

In addition to acting on the oocytes, Kit ligand is also involved in the interaction between the granulosa and theca cells due to the existence of Kit in the latter [18]. Kit ligand promotes the formation of theca layer by influencing the recruitment of theca cells in early follicle development [19], [20] and enhances proliferation of these cells in later stages, therefore influencing androgen production [20], [21]. Together with the Kit ligand-stimulated expression of aromatase in the granulosa cells, the increased androgen production from the theca cells would contribute to the output of estrogens from the follicle [12], [21], [22].

It has recently been reported that the Kit system in the zebrafish includes two forms of ligands (*kitlga* and *kitlgb*) and receptors (*kita* and *kitb*) due to the specific genome duplication in fish evolution, which is different from the situation in mammals [23]–[25]. Morpholino knockdown targeting the two ligands showed that the down-regulation of *kitlga* phenocopied the null allele of *kita* in controlling melanocyte development, suggesting that Kitlga might signal through Kita. However, *kitlgb* was not required [26]. In spite of this, there is a lack of direct evidence showing the interaction and specificity between Kit ligands and receptors and the role of the Kit system in the adult zebrafish, particularly in the ovary. Our recent expression analysis showed that both ligands and receptors displayed distinct temporal profiles during folliculogenesis and final oocyte maturation, suggesting differential roles for the Kit system in follicle development, particularly in the late stages [23]. One major issue that remains unknown is how the Kit system works in the zebrafish follicle, especially the spatial distribution of the two ligands and receptors and their binding specificity. To address this, we undertook this study by first investigating the spatial distribution of the Kit system (two ligands and two receptors) in the follicle followed by analyzing receptor specificity for both ligands and receptors using recombinant zebrafish Kitlga and Kitlgb produced by the Chinese hamster ovary (CHO) cells and receptors Kita and Kitb overexpressed in the COS cells. The discoveries were further confirmed by MAPK response to Kitlga and Kitlgb in both cultured zebrafish follicle cells and dechorionated mature oocytes. In addition, we attempted to elucidate the experimental data by theoretical modeling of the three-dimensional protein structures of the zebrafish Kit system. Finally, we performed oocyte maturation assay to verify the functional divergence of the two Kit ligand-receptor pathways. Our results provided strong evidence for a Kit-mediated bi-directional communication system in the zebrafish ovarian follicle, which could be part of the complex interplay between the oocyte and follicle cells during folliculogenesis.

## Materials and Methods

### Animals and chemicals

Zebrafish (*Danio rerio*) were obtained from a local tropical fish market and maintained in flow-through aquaria at 28±1°C on a photoperiod of 14L∶10D, with light on at 8:00. The fish was fed twice a day with the commercial tropical fish feed Otohime S1 (Marubeni Nisshin Feed Co., Tokyo, Japan) and once with frozen artemia. All experiments performed were licensed by the Government of the Hong Kong Special Administrative Region and endorsed by the Animal Experimentation Ethics Committee of The Chinese University of Hong Kong. Unless otherwise indicated, all common chemicals used were purchased from Sigma (St. Louis, MO), USB Corporation (Cleveland, OH), GE Healthcare (Waukesha, WI), or Merck (Whitehouse Station, NJ); enzymes from Promega (Madison, WI); and culture media from Gibco Invitrogen (Carlsbad, CA). Mouse KITL (mKITL) was purchased from Invitrogen (Carlsbad, CA). mKITL was first dissolved in water and then diluted to the desired concentrations with the medium before use. Antibodies for phospho-KIT (#sc-18076), HRP-linked anti-goat IgG (#sc-2056) and HRP-linked anti-rabbit IgG (#sc-2374) were from Santa Cruz Biotechnology (Santa Cruz, CA) and those for β-actin (#4967L), p44/42 MAP Kinase (#9102L) and phospho-p44/42 MAP Kinase (#9101L) were from Cell Signaling Technology (Danvers, MA).

### Construction of expression plasmids

Gene-specific primers (Table 1) flanking the open reading frame (ORF) of five genes (mouse *Kit*, zebrafish *kitlga*, *kitlgb*, *kita* and *kitb*) were used to amplify the ORF of each gene from the ovary of mouse or zebrafish. Restriction sites for *Hind* III and *Xho* I (for mouse *Kit*, zebrafish *kitlga*, *kitlgb* and *kita*) or *Not* I and *Xho* I (for zebrafish *kitb*) were respectively added to the 5′-end of the sense and antisense primers for subsequent cloning. The Kozak sequence (5′-GCCGCCACC-3′) was included in all sense primers before the start codon ATG to enhance translation efficiency [27]. PCR was performed for 36 cycles in a volume of 30 µl containing 10 µl template (RT reaction mix diluted at 1∶15), 1× PCR buffer, 0.2 mM each dNTP, 2.5 mM MgCl_2_, 0.2 mM each primer, and 1.5 U *Pfu* polymerase with the profile of 30 sec at 94°C, 30 sec at 60°C, and 4 min at 72°C. The PCR products were double digested with *Hind* III or *Not* I and *Xho* I and cloned into pCMV-Script vector (Stratagene, CA) for mouse *Kit*, zebrafish *kita* and *kitb* or pcDNA5/FRT vector (Invitrogen) for zebrafish *kitlga* and *kitlgb* at *Hind* III or *Not* I and *Xho* I sites downstream of the CMV promoter to generate five constructs: pCMV/mKIT, pCMV/zfKita, pCMV/zfKitb, pcDNA5/FRT/zfKitlga and pcDNA5/FRT/zfKitlgb. All the expression constructs were sequenced to confirm sequence fidelity. The sequencing reaction was performed with the BigDye Terminator Cycle Sequencing Kit v3.1 and analyzed on the ABI PRISM 3100 Genetic Analyzer (Applied Biosystems, Foster City, CA).

**Table 1:** Primers used in RT-PCR.

Gene	Strand	Sequence	Accession No.
*Kit*	Sense	GGCTGACTGTGCTGTGCTGATTG	NM_021099
	Sense	CTCACCTACAAATATTTGCAGAA	
	Antisense	CCCCTCGAGTCAGGCATCTTCGTGCACGA	
*kitlga*	Sense	AAGAAGCTTGCCGCCACCATGAATAATTCAAACATTTG	AY929068
	Antisense	CCCCTCGAGTTACACATCCATGATAATAT	
*kitlgb*	Sense	AATAAGCTTGCCGCCACCATGTTCCACATGAGGGAGGT	AY929069
	Antisense	CCTCTCGAGTTAGACCTCTGTGTCTGCAC	
*kita*	Sense	AATAAGCTTGCCGCCACCATGGAATATCACTGCGTTCT	NM_131053
	Antisense	CCCCTCGAGTCAGACTACAGGGTGACTTG	
	Antisense	CAAATATTTGTAGGTGAGCACAATCAGGATGAGAAC	
*kitb*	Sense	TATGCGGCCGCGCCGCCACCATGGGACACTCGTGGTTT	GQ994993
	Antisense	CCCCTCGAGTCAAGTAATCCTGCAAGATAC	
	Antisense	CAAATATTTGTAGGTGAGAACCACCAGAATGAAGCT	

To generate receptor chimeras containing the intracellular domain of mouse KIT (mKITID) and extracellular domain of zebrafish Kita or Kitb (zfKitaED or zfKitbED), the above antisense primer for mouse *Kit* and the sense primer corresponding to mKITID domain with an extra sequence complementary to the 3′-end of zfKitaED or zfKitbED were used in PCR to produce mKITID fragment. Similarly, the antisense primers corresponding to zfKitaED or zfKitbED domains with an extra sequence complementary to the 5′-end of mKITID and the above zebrafish *kita* or *kitb* sense primers were used to produce zfKitaED or zfKitbED fragments. The mKITID fragment was then mixed with zfKitaED or zfKitbED fragments and used as templates in PCR without primers to produce zfKitaED/mKITID or zfKitbED/mKITID. The chimeric products were then amplified with corresponding 5′- or 3′-end primers. After double digestion with *Hind* III and *Xho* I, the PCR products were cloned into pCMV-Script vector to generate pCMV/zfKitaED/mKITID and pCMV/zfKitbED/mKITID.

### Cell culture and transfection of COS-1 and Flp-In CHO cells

The primary follicle cell culture of zebrafish ovary was performed according to our previous report [28]. Briefly, the ovaries from about 20 female zebrafish were isolated and dispersed in a 100-mm petri dish containing 60% Leibovitz L-15 medium. The full-grown follicles were removed by sieving, followed by washing with medium M199 for five times. Afterwards, the follicles were cultured in M199 supplemented with 10% fetal calf serum (FCS) (Hyclone, Logan, UT) and antibiotics (streptomycin, 100 µg/ml; penicillin, 100 U/ml) at 28°C in 5% CO_2_ for 6 days for the proliferation of follicle cells. Then, the follicle cells were harvested by trypsinization and plated in 24-well plates at the density of about 2.5×10^5^ cells/well for 24 h. The cells were then starved with M199 without serum for 24 h before treatment.

The COS-1 cells were cultured in DMEM (Dulbecco Modified Eagle Medium) medium containing 10% FCS and antibiotics at 37°C with 5% CO_2_ [29]. The expression plasmids pCMV/mKIT, pCMV/zfKita, pCMV/zfKitb, pCMV/zfKitaED/mKITID and pCMV/zfKitbED/mKITID were transfected into the COS-1 cells with Lipofectamine 2000 (Invitrogen) according to the manufacturer's protocol. The expression vector pCMV-Script was used as the control. Twelve hours after transfection, the cells were subcultured into 24-well plates at the density of 10^5^ cells/well. The medium was changed to serum-free medium after 24-h incubation, and the incubation continued for another 24 h before drug treatment.

The Flp-In CHO cells (Invitrogen) were cultured in Ham F-12 medium containing 10% FCS and antibiotics at 37°C with 5% CO_2_. The cells were subcultured in 10-cm culture dishes (Falcon, Franklin Lakes, NJ) and allowed to grow to 25% confluence before transfection. The expression constructs pcDNA5/FRT/zfKitlga and pcDNA5/FRT/zfKitlgb (0.5 µg) were cotransfected into the CHO cells separately using Lipofectamine 2000 (Invitrogen) together with 4.5 µg of plasmid pOG44 (Invitrogen) that encodes a recombinase to facilitate homologous recombination of the expression plasmids at the specific FRT site.

### Production of recombinant Kitlga and Kitlgb

The CHO cells transfected with expression constructs, pcDNA-Kitlga or pcDNA-Kitlgb, were selected by hygromycin B (Invitrogen) at 500 µg/ml for one month and then diluted serially to 96-well plates at a density of 2–3 cells/well. After about one week, the wells containing single colonies were labeled and the colonies were further expanded in 100-mm petri dishes until the cells grew to half confluence. Each clone was subject to RNA extraction for real-time qPCR and Northern blot hybridization for expression as well as PCR analysis of genomic DNA for plasmid integration. The positive clones were then used to produce recombinant proteins according to the protocol described in our previous report [30]. Briefly, the cells were subcultured into 750 ml flasks (Falcon) in 50 ml Ham F12 medium supplemented with 10% FCS and allowed to grow to about 90% confluence at 37°C. The FCS-containing medium was then replaced with 50 ml FCS-free medium and the culture temperature was reduced to 28°C. After a further 5-day incubation, the medium was harvested and concentrated by 200 folds with the Labscale TFF System with Pellicon XL Biomax 8 filter (Millipore, Bedford, MA).

### Total RNA isolation and RT

Total RNA was extracted from cultured follicle cells with Tri-Reagent (Molecular Research Center, Cincinnati, OH) according to the manufacturer's protocol and our previous study [28]. RT was then performed at 37°C for 2 h in a volume of 10 µl containing 0.5 µg of oligo(dT), 1× M-MLV RT buffer, 0.5 mM each deoxyribonucleotide triphosphate (dNTP), 0.1 mM dithiothreitol, and 100 U of M-MLV RT (Invitrogen).

### Separation of oocytes and follicle layers

The method of separating oocytes and follicle layers was based on our previous report [31]. In brief, the full-grown follicles were pretreated in Cortland's medium without calcium and magnesium ions but with EDTA (1 mM) for about one hour to reduce the cohesion between the oocyte and the follicle layer. The follicle layer was then carefully peeled off with fine forceps without damaging the oocyte. The isolated follicle layers and the denuded oocytes from five follicles were pooled and subject to RT-PCR analysis.

### Isolation and dechorionation of ovulated eggs

To collect ovulated eggs, fish with similar body size were placed (8 males and 8 females) in a tank one day before follicle collection. The ovaries collected at 0800 h were quickly dispersed in Cortland's medium, and ovulated eggs were isolated and transferred into 24-well plate (about 20 eggs per well). Trypsin (0.25%) was added into wells for dechorionation of ovulated eggs. After 20 min-incubation with trypsin, 1% ovomucoid (Worthintgon, Lakewood, NJ) was added to inactivate trypsin. After washing with Cortland's medium, the dechorionated eggs were then subjected to drug treatment.

### Oocyte maturation assay

Full-grown follicles of around 0.65 mm in diameter were isolated and placed in Cortland's medium as previously reported [32]. The healthy follicles were selected and randomly distributed into a 24-well plate with 40 follicles per well in a total of 400 µl medium. The follicles were pretreated with recombinant media or control medium at 28°C for 6 h followed by treatment with 17α, 20β-dihydroxy-4-pregnen-3-one (DHP, 5 ng/ml). After 9 h of incubation, the follicles that had undergone GVBD, a marker for oocyte maturation, were scored.

### Northern blot analysis

Northern blot hybridization was performed according to our previous report [33]. In brief, total RNA (20 µg) from the cells was separated in a 1% denaturing agarose gel containing 2.2 M formaldehyde, transferred to a positively charged nylon membrane (Roche, Mannheim, Germany), and UV cross-linked with the GS Gene Linker (Bio-Rad, Hercules, CA). The membrane was then hybridized with DIG-labeled cRNA probes in vitro transcribed from the cloned zebrafish *kitlga* or *kitlgb* cDNA, detected with the Chemiluminescent Detection Kit according to the manufacturer's protocol (Roche), and analyzed on the Lumi-Imager F1 Workstation (Roche).

### Quantification of gene expression by real-time qPCR

Real-time qPCR was performed to quantify the expression of *kitlga* and *kitlgb* in cloned CHO cells according to our previous report [23]. Briefly, the template for the standard curve was prepared by PCR amplification of cDNA fragment with specific primers, and the copy numbers of the DNA molecules were calculated before use as templates to construct standard curves in real-time qPCR. All PCR reactions were performed in a total volume of 30 µl containing 10 µl template (RT reaction mix diluted at 1∶15), 1× PCR buffer, 0.2 mM each dNTP, 2.5 mM MgCl_2_, 0.75 U of Taq polymerase, 0.5× EvaGreen (Biotium, Hayward, CA), and 20 nM fluorescein (Bio-Rad) on the iCycler iQ Real-time PCR Detection System (Bio-Rad). The amplification protocol was 30 sec at 94°C, 30 sec at 60°C, and 30 sec at 72°C, with a signal detection period of 7 sec at 80°C. A melt curve analysis was performed at the end of the reaction to check the reaction specificity.

### Western blotting

The cells or follicles were lysed by adding 1× SDS sample buffer (62.5 mM Tris-HCl, pH 6.8 at 25°C, 1% w/v SDS, 10% glycerol, 5% 2-mercaptoethanol, 100 µl per well of 24-well plate). Then the plate was shaken immediately for a few times and the extract from each well transferred to a microcentrifuge tube. All samples were heated to 95–100°C for 5 min, cooled on ice, and microcentrifuged for 5 min. Western blotting was performed according to the manufacturer's protocol (Cell Signaling Technology). Briefly, samples (half from each well of the 24-well plate) were loaded and separated in the 12.5% SDS–PAGE gel in 1× running buffer (25 mM Tris base, 0.2 M glycine, 0.1% w/v SDS), followed by blotting to the nitrocellulose membrane (Bio-Rad) using blotting buffer (25 mM Tris base, 0.2 M glycine, 20% methanol). The membrane was incubated in 25 ml blocking buffer (1× TBS, 0.1% Tween-20,and 5% w/v nonfat dry milk) for 1 h at room temperature and then incubated in 5 ml of diluted primary antibody (1∶1000 in blocking buffer) at 4°C overnight. The membrane was washed three times for 5 min each with wash buffer (1× TBS, 0.1% Tween-20) and then incubated with HRP-conjugated secondary antibody (1∶2000 in wash buffer) for 1 h at room temperature. The membrane was then washed again and equilibrated with the developing solution (Western Blotting Luminol Reagent; Santa Cruz Biotechnology). The signals were detected on the Lumi-Imager F1 Workstation (Roche).

### Molecular modeling

The three-dimensional structures of zebrafish Kit ligands and receptors were generated on the basis of the known crystal structures of human KITLG and KIT (PDB code 2E9W) and mouse KITL and KIT (PDB code 2O26) using the SWISS-MODEL automated homology modelling server of the Swiss Institute of Bioinformatics [34], [35]. The structures were visualized using the Swiss-PdbViewer program available from the ExPASy Server [36].

### Data analysis

The ratio of phosphorylation levels to that of the internal control β-actin (*bactin*, Bactin) was calculated and then expressed as the fold change compared to the control. All values were expressed as the mean ± SEM and the data were analyzed by one-way ANOVA followed by Dunnett test using Prism 5 on Macintosh OS X (GraphPad Software, San Diego, CA).

## Results

### Spatial distribution of Kit system within the follicle

**Figure 1:**
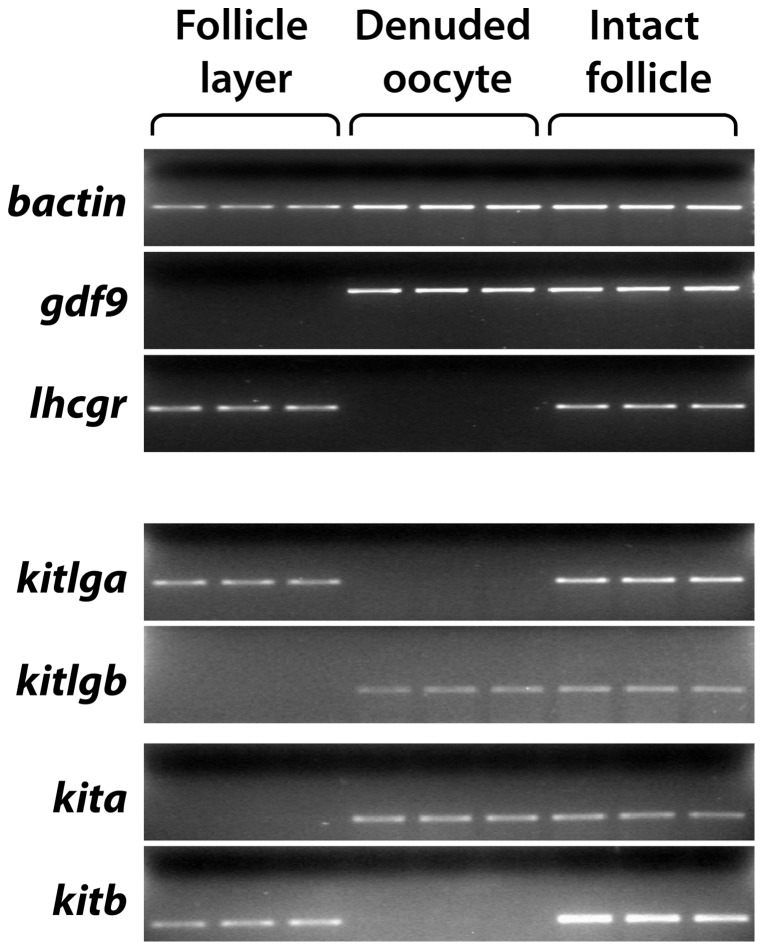
Distribution of the Kit system within the zebrafish follicle. The somatic follicle layer was separated from the oocyte followed by RNA extraction and semi-quantitative PCR detection in the two compartments (follicle layer and denuded oocyte). Each sample represents the total RNA pooled from 5 follicles. The housekeeping gene *bactin* was used as the internal control for all samples, whereas *gdf9* and *lhcgr* were used as the markers for denuded oocytes and somatic follicle layers, respectively.

Zebrafish ovarian follicle consists of two compartments: a developing oocyte in the center and a surrounding thin follicle layer of somatic cells containing the granulosa and theca cells. To understand the action modes of Kitlga and Kitlgb within the follicle, it is essential to find out the relative distribution of the two ligands and their potential receptors Kita and Kitb in the two compartments. We therefore performed the experiment by mechanically separating the follicle layer from the oocyte followed by examining the expression of Kit ligands and receptors in the two compartments. To ensure a clean separation of the two compartments, we used a gonadotropin receptor, *lhcgr*, and *gdf9* as the molecular markers for the follicle layer and oocyte, respectively. Consistent with our previous reports [37], *lhcgr* was exclusively expressed in the somatic follicle cells whereas the expression of *gdf9* was restricted in the oocyte. Interestingly, *kitlga* was only expressed in the follicle cells and *kita* only in the oocyte, which is consistent with the situation in mammals. However, *kitb* was exclusively expressed in the follicle layer and *kitlgb* only in the oocyte, which differs from that in mammals (Fig. 1).

### Mouse KITL activation of mouse KIT but not zebrafish Kita and Kitb

**Figure 2:**
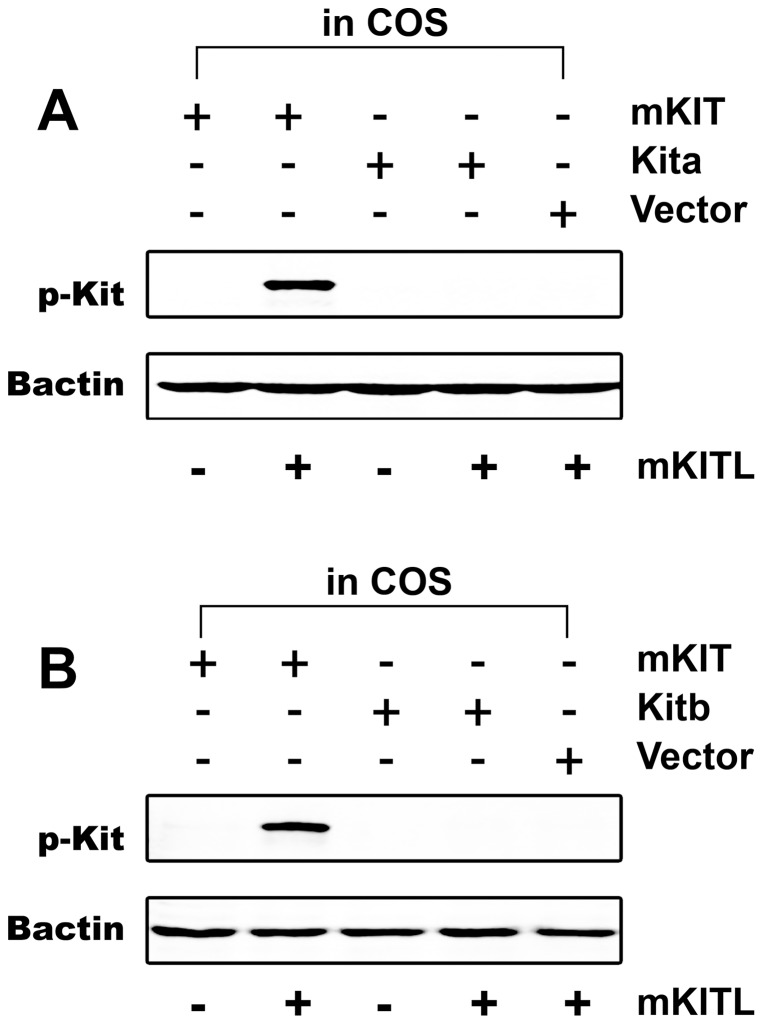
Species specificity of Kit activation by Kit ligand. Plasmids expressing mouse KIT (mKIT), zebrafish Kita (A) or Kitb (B) were transfected into the COS-1 cells. After treatment with recombinant mouse KITL (mKITL) for 10 min, the phosphorylation of Kit was detected by an antibody recognizing the tyrosine phosphorylation site in the intracellular domain. Plasmid pCMV-Script (Vector) was used as a negative control. Bactin was used as the internal control for all samples. p-Kit, Kit phosphorylation; +, recombinant ligand proteins were added or cells were transfected with specific receptor plasmids; −, no recombinant ligands or transfection with receptor plasmids.

Considering that recombinant zebrafish Kit ligands were not available commercially, we first tested whether mouse KITL could be used to replace zebrafish Kit ligands for further functional study. As a positive control, the COS-1 cells transfected with the plasmid expressing mouse KIT protein were treated with mouse KITL. A strong KIT phosphorylation signal was observed by Western blot analysis, which demonstrated the feasibility of our experimental system. Zebrafish Kita and Kitb, however, had no significant response to mouse KITL (Fig. 2), suggesting that the mammalian molecule is not functional in the zebrafish system.

### Production of recombinant zebrafish Kitlga and Kitlgb

**Figure 3:**
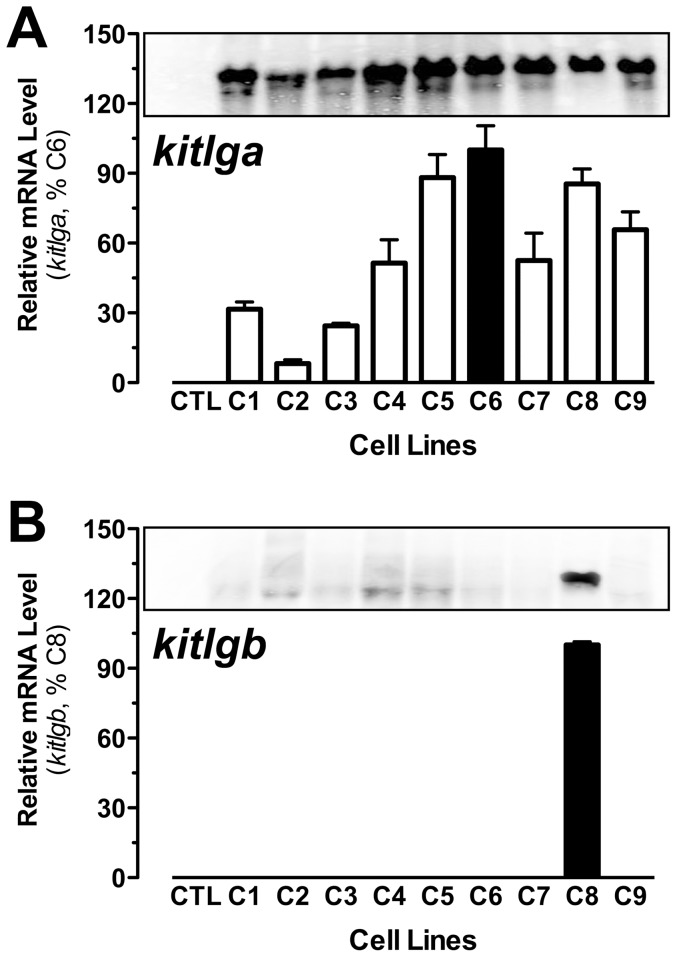
Characterization of recombinant CHO clones expressing zebrafish Kitlga and Kitlgb. Nine clones (C1–9) for Kitlga (A) and one clone (C8) for Kitlgb (B) were chosen for analysis by real-time qPCR (bottom) and Northern blot hybridization (top). CTL, control CHO cells transfected with the vector pcDNA5/FRT.

To study the biological functions of the Kit system in the zebrafish ovary,including receptor specificity of Kitlga and Kitlgb and their differential roles in the follicle, the availability of zebrafish Kitlga and Kitlgb is indispensable. Based on our previous work in producing recombinant zebrafish gonadotropins [30] and goldfish follistatin [38], we chose the Flp-In CHO cell system to produce recombinant zebrafish Kitlga and Kitlgb. After transfection, hygromycin B selection, and cloning by serial dilution, we obtained a number of stably-transfected clones. Some of these clones (C1–C9) were subject to further characterization by both real-time qPCR and Northern blot analysis for gene expression. As demonstrated in Fig. 3, all selected clones for *kitlga* exhibited high levels of expression although the relative abundance of mRNA varied among these clones. In contrast, only one clone was identified to express *kitlgb* (Fig. 3). This *kitlgb*-positive clone (C8) and the *kitlga* clone with the maximal expression level (C6) were chosen for large-scale production of recombinant Kitlga and Kitlgb according to our previous report [30].

### Receptor specificity of zebrafish Kit ligands and receptors

**Figure 4:**
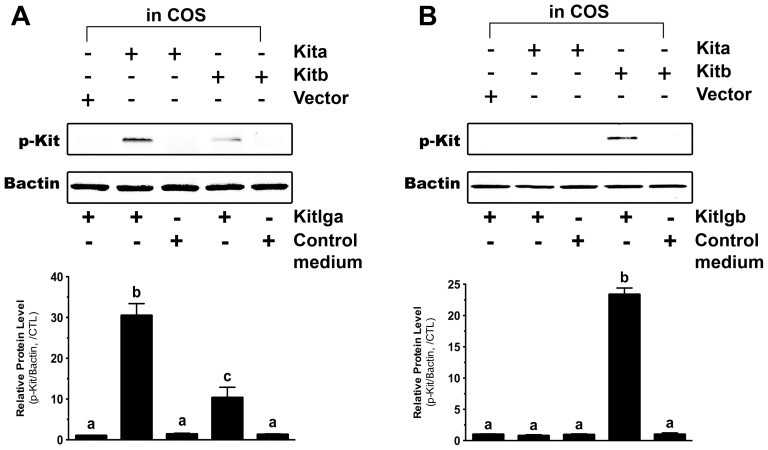
Response of zebrafish Kita and Kitb to zebrafish Kitlga and Kitlgb. The COS-1 cells expressing Kita and Kitb were treated with conditioned media containing recombinant Kitlga (A) and Kitlgb (B) for 10 min followed by Western blot analysis for Kit phosphorylation. The control medium was from the CHO cells carrying the control plasmid pcDNA5/FRT. The expression plasmid pCMV-Script (Vector) was used as the control in the COS-1 cells. The densitometric analysis of the Western signals is shown at the bottom of each graph. The data were normalized to Bactin and expressed as the fold change compared to the first group (mean ± SEM, n = 3). Different letters indicate statistical significance (*P*<0.05). p-Kit, Kit phosphorylation; +, recombinant ligand proteins were added or cells were transfected with specific receptor plasmids; −, no recombinant ligands or transfection with receptor plasmids.

**Figure 5:**
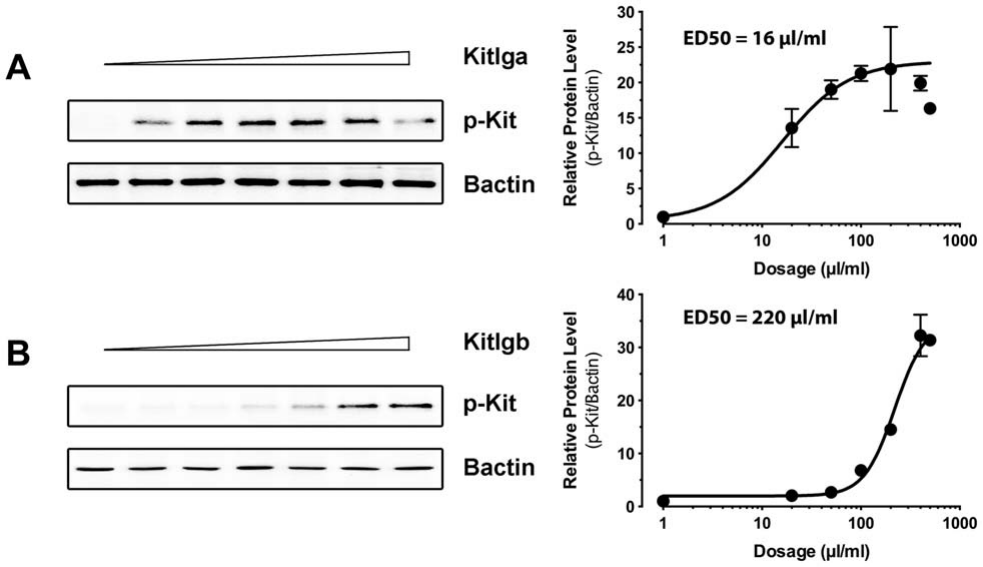
Dose response activation of Kita and Kitb by Kit ligands. The COS-1 cells expressing Kita and Kitb were treated with conditioned media containing recombinant Kitlga (A) and Kitlgb (B) for 10 min followed by Western blot analysis for Kit phosphorylation (left) and characterization of Kitlga and Kitlgb potency (right). The ED50 for each ligand was defined as 1 Unit. p-Kit, Kit phosphorylation.

The conditioned media containing recombinant zebrafish Kitlga and Kitlgb were then used to treat the COS-1 cells transfected with the plasmids expressing receptor protein Kita or Kitb. As shown by receptor phosphorylation, Kitlga preferentially activated Kita. Although it also activated Kitb, the potency was much less than that on Kita. Treatment with Kitlga produced no signals in the cells transfected with the control plasmid. Also, the control medium from the cell line carrying the vector only had no effect on the activation of either Kita or Kitb (Fig. 4A). On the other hand, recombinant zebrafish Kitlgb specifically activated Kitb without any detectable effect on Kita (Fig. 4B). Dose response experiment showed that both Kitlga and Kitlgb activated their preferred receptors Kita and Kitb in a clear dose-dependent manner. The conditioned media containing recombinant Kitlga and Kitlgb appeared to have different potencies with the ED50s of Kitlga and Kitlgb being about 16 µl/ml and 220 µl/ml, respectively (Fig. 5). For future functional studies, we will define these doses as one unit (U).

**Figure 6:**
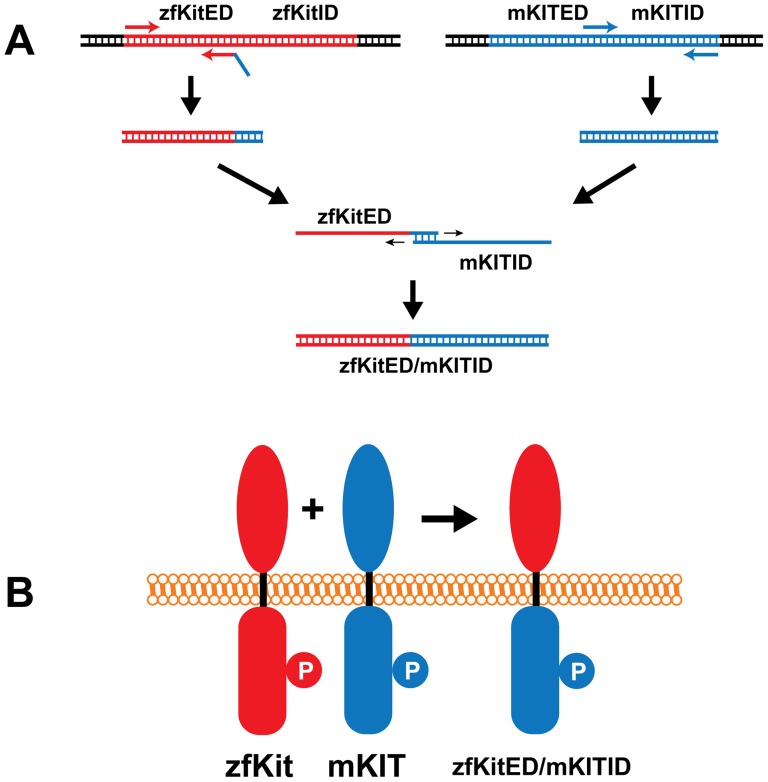
Schematic illustration of plasmid construction for expressing chimeric receptors. The mouse Kit receptor is shown in blue and zebrafish Kita or Kitb is in red. The extracellular domain of zebrafish Kita or Kitb (zfKitED) and intracellular domain of mouse KIT (mKITID) were amplified by PCR followed by extension of the PCR products on each other to generate a DNA fragment (A) coding for the fusion protein zfKitED/mKITID (B). “P” represents the phosphorylated tyrosine site in the intracellular domain that is recognized by the antibody.

**Figure 7:**
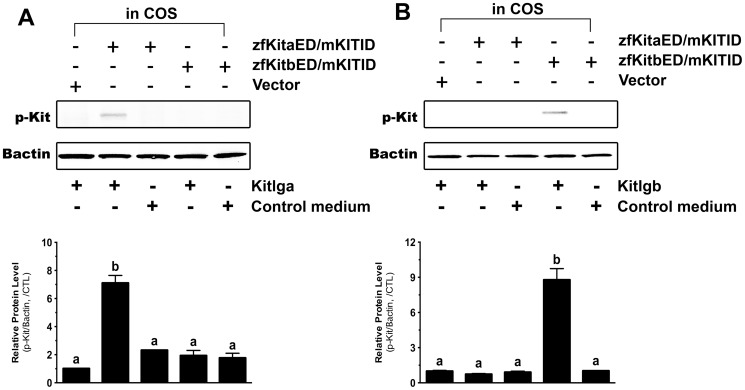
Response of the chimeric zebrafish/mouse Kit receptors to zebrafish Kitlga and Kitlgb. The COS-1 cells expressing zfKitaED/mKITID and zfKitbED/mKITID were treated with recombinant Kitlga (A) and Kitlgb (B) for 10 min followed by Western blot analysis for Kit phosphorylation. The control medium was from the CHO cells carrying the control plasmid pcDNA5/FRT. The expression plasmid pCMV-Script (Vector) was used as the control in the COS-1 cells. The densitometric analysis of the Western signals is shown at the bottom of each graph. The data were normalized to Bactin and expressed as the fold change compared to the first group (mean ± SEM, n = 3). Different letters indicate statistical significance (*P*<0.05). p-Kit, Kit phosphorylation; +, recombinant ligand proteins were added or cells were transfected with specific receptor plasmids; −, no recombinant ligands or transfection with receptor plasmids.

To further confirm the ligand-receptor specificity demonstrated above with the antibody against mammalian Kit, we examined the receptor specificity by using two receptor chimeras that contained the intracellular domain of mouse KIT and extracellular domains of zebrafish Kita or Kitb (zfKitaED/mKITID and zfKitbED/mKITID), respectively (Fig. 6). As shown in Fig. 7, zfKitaED/mKITID responded specifically to Kitlga but not Kitlgb. In contrast, Kitlgb specifically activated zfKitbED/mKITID but not zfKitaED/mKITID.

### Effects of Kitlga and Kitlgb on MAPK phosphorylation in cultured follicle cells

**Figure 8:**
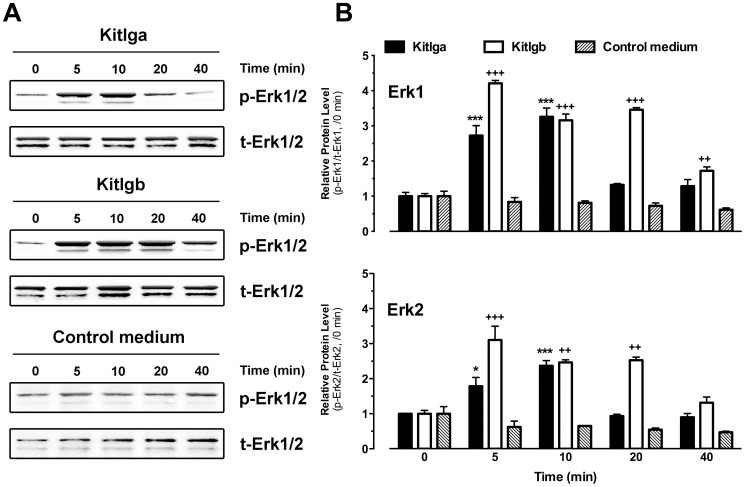
Effects of Kitlga and Kitlgb on MAPK phosphorylation in cultured follicle cells. (A) Western blot analysis of MAPK (Erk1/2) phosphorylation in response to Kitlga (200 µl Kitlga conditioned medium+200 µl control medium per ml), Kitlgb (400 µl/ml Kitlgb conditioned medium) and control medium (400 µl per ml). (B) Densitometric quantification of Erk1 (upper) and Erk2 (lower) phosphorylation. The data were normalized to total Erk1 or Erk2 and expressed as the fold change compared to the first group at time 0 min (mean ± SEM, n = 3). */+ P<0.05; **/++ P<0.01; ***/+++ P<0.001. p-Erk1/2, phosphorylated Erk1/2; t-Erk1/2, total Erk1/2.

As documented in mammalian models, the binding of KITL to KIT leads to the activation of many intracellular signaling pathways including MAPK [39]. As shown earlier, zebrafish Kita and Kitb were exclusively expressed in the oocyte and follicle cells, respectively. After demonstrating receptor specificity of the zebrafish Kit system, we performed a further experiment to confirm the biological activity of recombinant Kitlga and Kitlgb in cultured zebrafish follicle cells by examining MAPK phosphorylation in response to the recombinant ligands. A concentration that produced maximal effect in activating Kit receptors was used for the two ligands (for Kitlga treatment, 200 µl Kitlga conditioned medium+200 µl control medium per ml; for Kitlgb treatment, 400 µl/ml Kitlgb conditioned medium). Both Kitlga and Kitlgb significantly activated MAPK (Erk1/2) in a time-dependent manner in comparison with the constant phosphorylation level of MAPK in the cells treated with the control medium. Different from Kitlga, Kitlgb showed stronger and more persistent effect on MAPK phosphorylation in the follicle cells where Kitb was expressed (Fig. 8).

### Effects of Kitlga and Kitlgb on MAPK phosphorylation in ovulated oocytes

To further confirm the location of Kita but not Kitb in the oocyte, we examined MAPK phosphorylation in response to Kitlga and Kitlgb in ovulated oocytes, which were free of the follicle cells.

**Figure 9:**
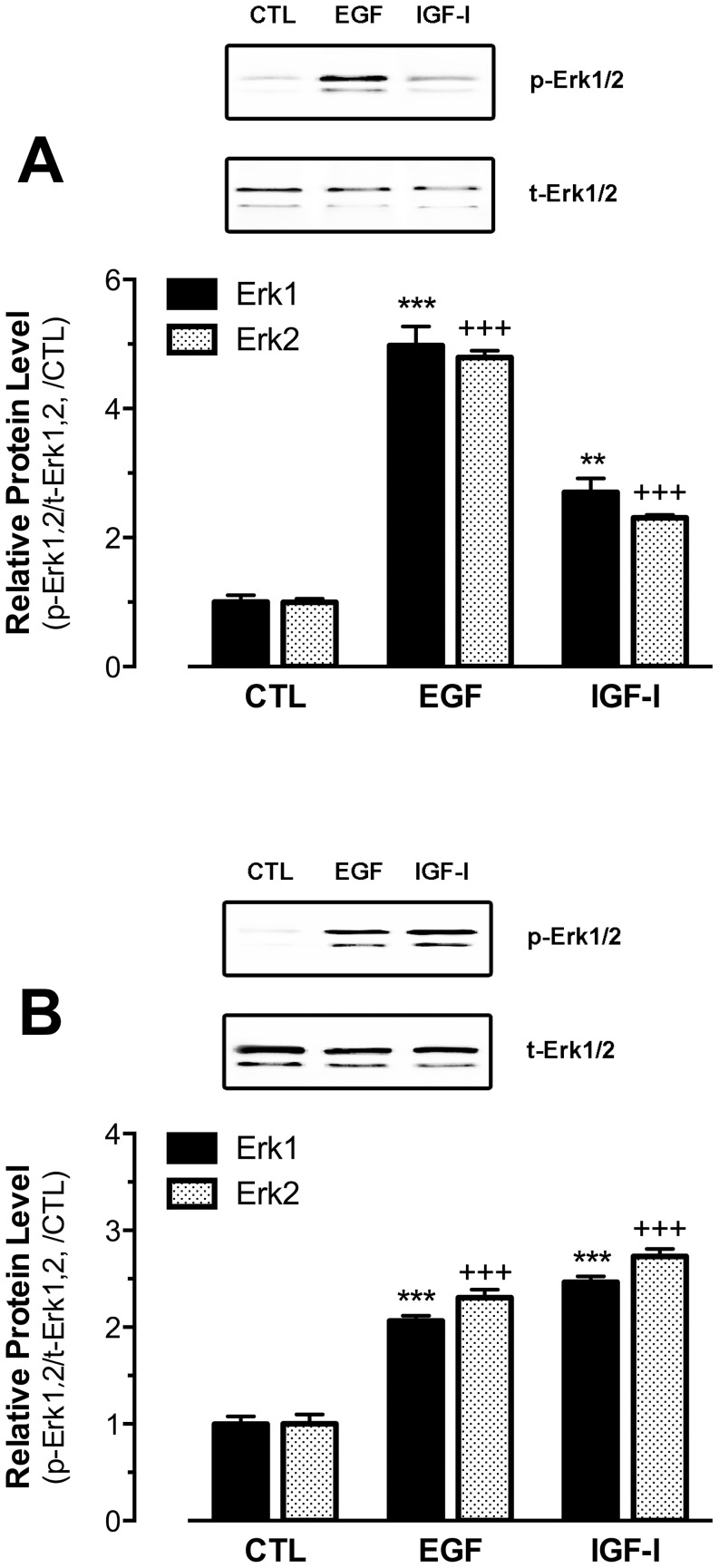
Effects of EGF and IGF-I on MAPK phosphorylation in immature intact follicles or ovulated mature oocytes. The immature intact full-grown follicles (A) or ovulated mature oocytes (B) were treated with human EGF or IGF-I for 10 min followed by Western blot analysis for MAPK (Erk1/2) phosphorylation. The Western blot image is shown on the top of each graph and the densitometric quantification is at the bottom. The data were normalized to the total Erk1 or Erk2 and expressed as the fold change compared to the control (CTL) (mean ± SEM, n = 3). **/++ P<0.01; ***/+++ P<0.001. p-Erk1/2, phosphorylated Erk1/2; t-Erk1/2, total Erk1/2.

As a control to test the functionality of the assay, we first examined the responsiveness of the intact full-grown immature follicles to epidermal growth factor (EGF) and insulin-like growth factor I (IGF-I) as EGF and IGF-I are well known to activate MAPK through their receptors, EGFR and IGF-IR, respectively [40], [41]. Previous studies have demonstrated the expression of EGFR (*egfr*) in the zebrafish follicle cells [31] and IGF-IR (*igf1ra* and *igf1rb*) in both follicle cells and oocytes [42]. As shown in Fig. 9A, both growth factors significantly activated MAPK and EGF was more potent than IGF-I. We then treated the mature and ovulated oocytes with the same factors. Interestingly, both factors could activate MAPK in the follicle cell-free mature and ovulated oocytes, but IGF-I appeared to be more potent than EGF in activating MAPK phosphorylation, different from their potencies in the full-grown intact follicles (Fig. 9B).

**Figure 10:**
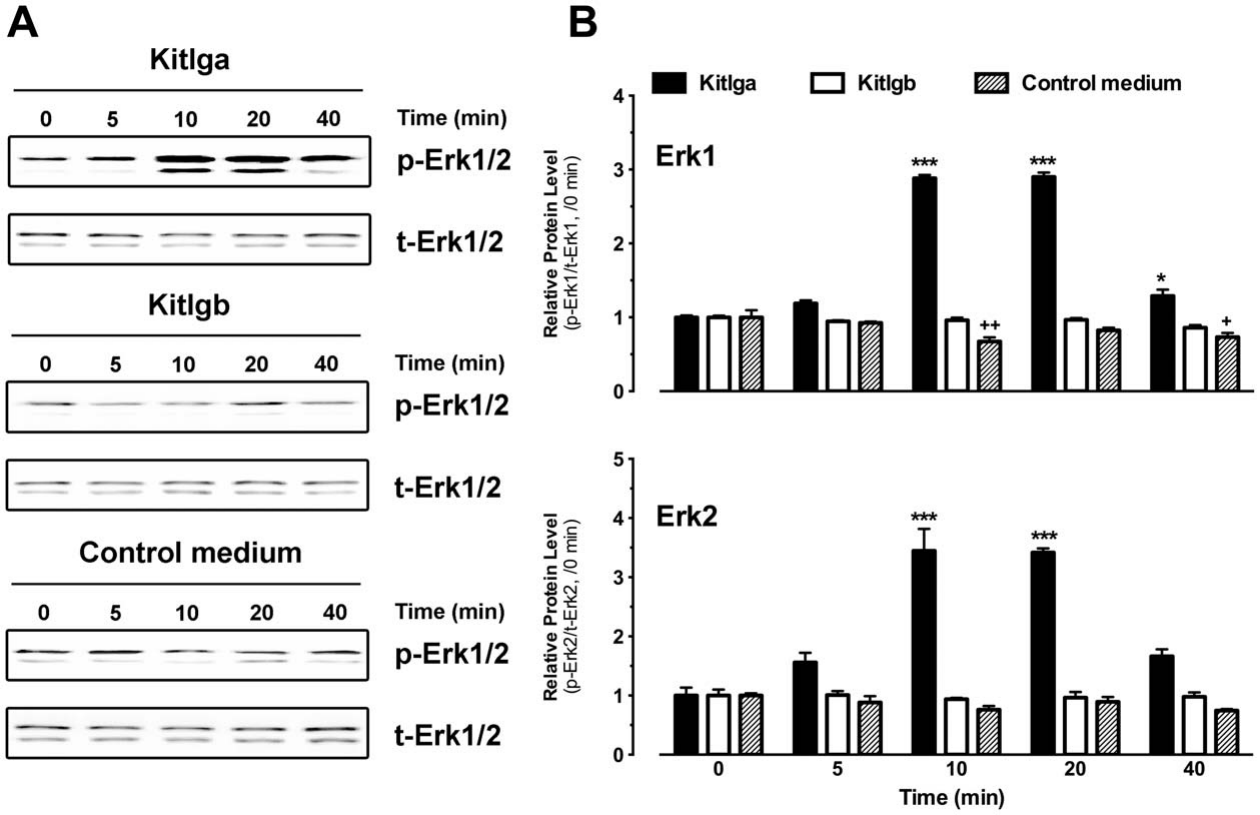
Effects of Kitlga and Kitlgb on MAPK phosphorylation in mature and ovulated oocytes. (A) Western blot analysis of MAPK (Erk1/2) phosphorylation in response to Kitlga (200 µl Kitlga conditioned medium+200 µl control medium per ml), Kitlgb (400 µl/ml Kitlgb conditioned medium) and control medium (400 µl per ml). (B) Densitometric quantification of Erk1 (upper) and Erk2 (lower) phosphorylation. The data were normalized to total Erk1 or Erk2 and expressed as the fold change compared to the first group at time 0 min (mean ± SEM, n = 3). */+ P<0.05; ***/+++ P<0.001. p-Erk1/2, phosphorylated Erk1/2; t-Erk1/2, total Erk1/2.

Subsequently, we treated the ovulated oocytes with recombinant zebrafish Kitlga (200 µl Kitlga+200 µl control medium per ml) or Kitlgb (400 µl/ml). As shown in Fig. 10, Kitlga increased MAPK phosphorylation level in the oocytes in a time-dependent manner. The effect turned significant at 10 min of treatment and lasted up to 40 min. In contrast, Kitlgb, whose receptor Kitb was not present in the oocyte, had no effect on MAPK (Fig. 10).

### Structural analysis for binding specificity between zebrafish Kit ligands and receptors

**Figure 11:**
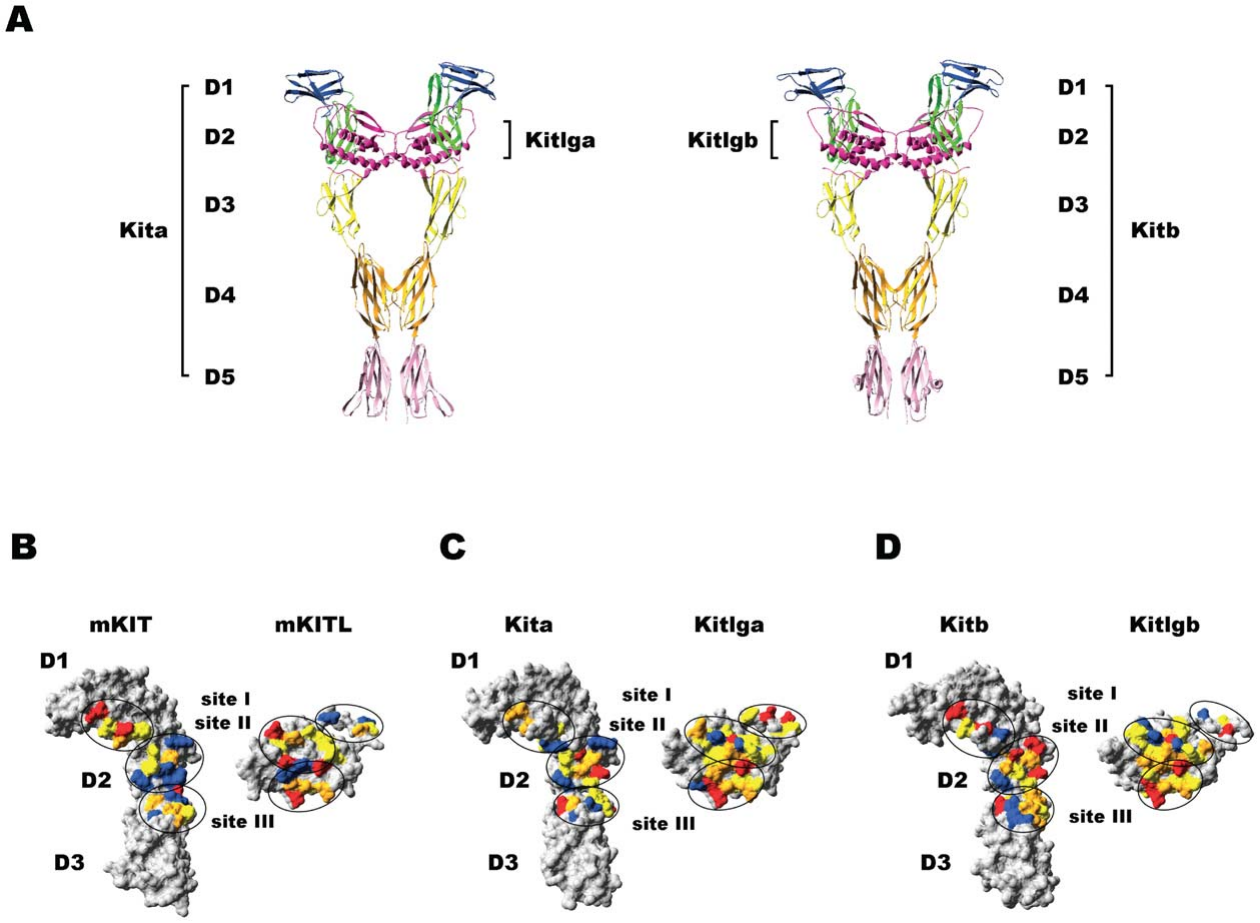
Molecular modeling of the three-dimensional structures of zebrafish Kit ligands (Kitlga and Kitlgb) and receptors (Kita and Kitb). (A) Ribbon model of the Kitlga-Kita (left) and Kitlgb-Kitb (right) complexes. The motif D1 is colored in blue, D2 in green, D3 in yellow, D4 in orange, and D5 in pink. Kit ligands are colored in magenta. (B–D) Surface models of the interphase of the mouse KITL-KIT (B), zebrafish Kitlga-Kita (C) and Kitlgb-Kitb (D) complexes. The Kit receptor part involving D1, D2 and D3 is shown on the left and the ligand part is on the right. The amino acids with negative charge, positive charge, polar and hydrophobic side chains are shown in red, blue, orange and yellow, respectively. The ligand-receptor binding sites (site I, site II, and site III) are circled.

To elucidate the mechanism underlying the receptor binding specificity described above (Kitlga for Kita and Kitlgb for Kitb), three-dimensional structures of zebrafish Kit ligands and receptors and their binding sites were analyzed. Homology modeling using the known crystal structures of human KITLG and KIT and mouse KITL and KIT as templates demonstrated the conserved three-dimensional protein structures of zebrafish Kit system (Fig. 11A). Both extracellular domains of zebrafish Kita and Kitb contained five immunoglobulin (Ig)-like motifs designated D1, D2, D3, D4 and D5, and both ligands were classic four-helix bundle type cytokines, which were fully consistent with their corresponding mammalian counterparts [43], [44]. Three binding sites were identified at the Kit ligand-Kit receptor interface in the zebrafish Kit system according to structure-based alignments of zebrafish, mouse and human Kit ligands or receptors (Fig. 11B).

**Figure 12:**
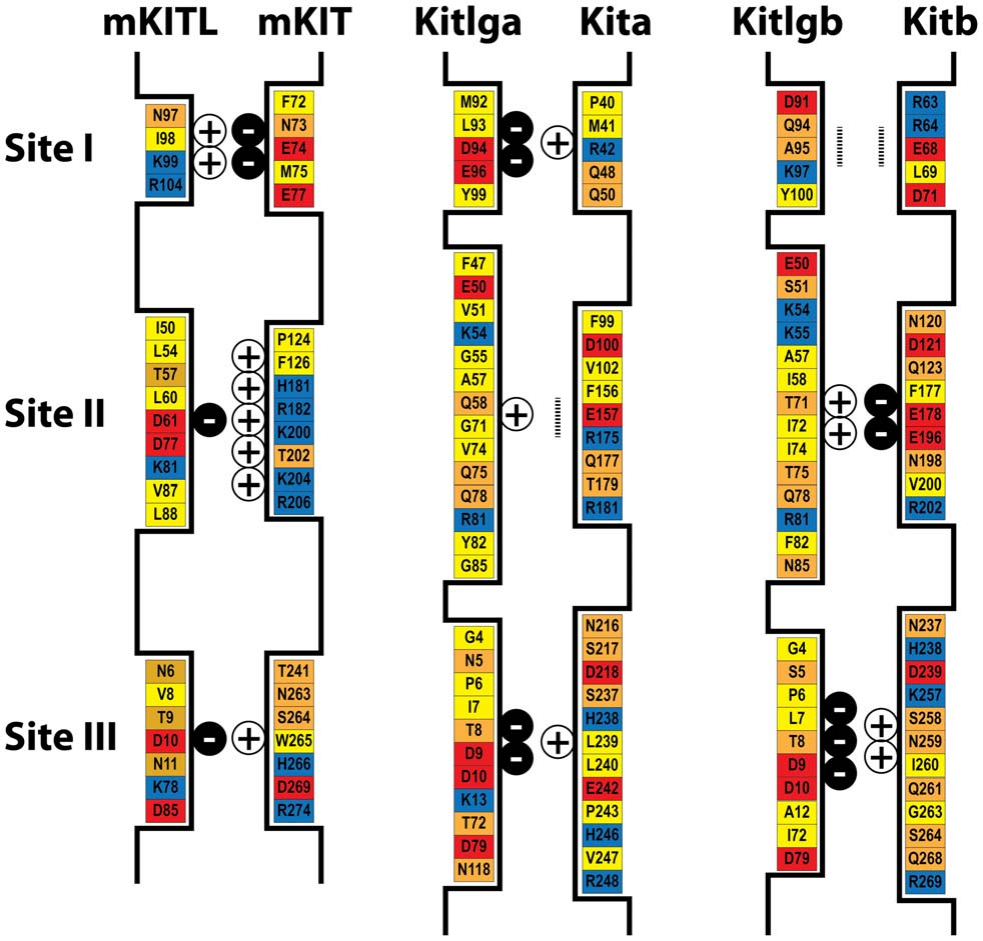
Schematic illustration of the three ligand-receptor binding sites and the amino acids involved in the mouse and zebrafish. The amino acids with negative charge, positive charge, polar and hydrophobic side chains are shown in red, blue, orange and yellow, respectively. The amount of net charges at each site is indicated by plus or minus signs, and the vertical dotted bar indicates neutral site.

As reported in previous studies [7], [45], charge complementarity appeared to be an important factor for the interaction between Kit ligand and receptor. Mouse KIT was positively charged at both site II and III, but negatively charged at site I; in contrast, mouse KITL showed opposite and complementary pattern of charge distribution with site II and III being negatively charged and site I positively charged (Fig. 12). In comparison with the charge distribution and complementarity in the mouse, zebrafish Kitlga was negatively charged at site I and III and slightly positively charged at site II, whereas Kitlgb was rich in negative charge at site III and positive charge at site II. The site I of Kitlgb appeared to be neutral due to the existence of equal negative and positive charges. As for receptors, Kita was positively charged at both site I and III and neutral at site II; in contrast, Kitb was rich in negative charge at site II and positive charge at site III, but neutral at site I. In both mouse and zebrafish, the site II and III are more abundant in uncharged amino acids in both the ligands and receptors compared with site I (Fig. 12).

### Diverse functions of two Kit ligand-receptor pathways in the zebrafish ovary

**Figure 13:**
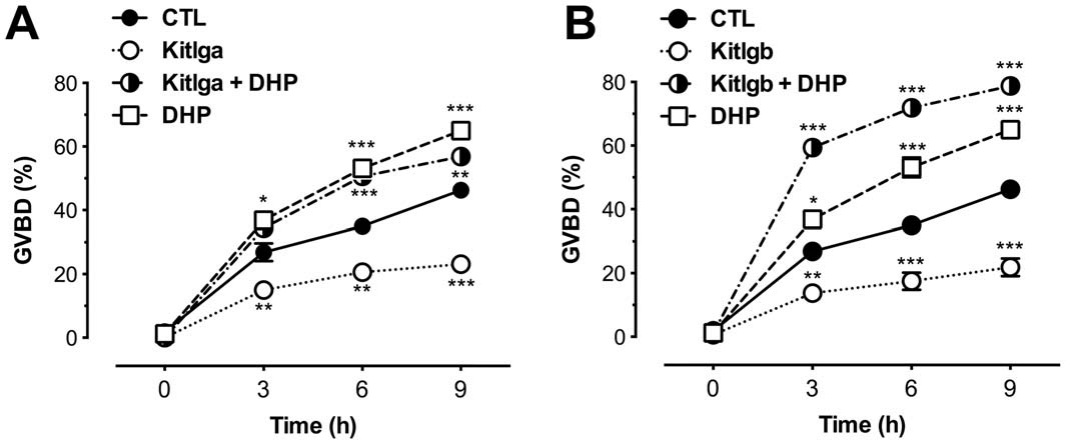
Effects of recombinant zebrafish Kitlga and Kitlgb on oocyte maturation. Full-grown immature follicles were pretreated for 6 h with Kitlga (200 µl Kitlga conditioned medium+200 µl control medium per ml), Kitlgb (400 µl/ml Kitlgb conditioned medium) and control medium (CTL, 400 µl per ml)), respectively, followed by treatment with DHP (5 ng/ml) for 9 h. Germinal vesicle breakdown (GVBD) was scored at different time points and expressed as the percentage (mean ± SEM, n = 4).

Our experimental and theoretical modeling data both suggested the existence of two different pathways, Kitlga-Kita and Kitlgb-Kitb, in the zebrafish follicle. To provide further evidence for differential functions of the two pathways, we performed oocyte maturation assay to test recombinant zebrafish Kitlga and Kitlgb. Full-grown immature follicles were pretreated with Kitlga (200 µl Kitlga conditioned medium+200 µl control medium per ml), Kitlgb (400 µl/ml Kitlgb conditioned medium) or control medium (400 µl/ml) for 6 h, followed by a 9-h incubation with DHP, the maturation-inducing hormone. The follicles were then scored for germinal vesicle breakdown (GVBD). As shown in Fig. 13, both Kitlga and Kitlgb significantly reduced the basal rate of GVBD compared with the control group. Interestingly, Kitlgb significantly raised the rate of GVBD in the presence of DHP; in contrast, Kitlga had no effect on DHP-induced GVBD.

## Discussion

In the past decade, accumulating evidence demonstrates that, instead of being a passive recipient, the oocyte plays an essential role in directing the development of ovarian follicles throughout folliculogenesis from the initial assembly of the primordial follicles to ovulation. It is now generally accepted that there exist extensive bi-directional communications between the oocytes and follicle cells, particularly the granulosa cells [46]. A complex interplay of regulatory factors from both the follicle cells and oocytes governs the process of folliculogenesis.

Our previous work has demonstrated the existence of an intimate bi-directional communication network between the oocyte and its surrounding follicle layer in the zebrafish ovary [47]. EGF and activin families represent two major groups of ovarian factors involved in this process [31]. The ligands of EGF family including EGF (*egf*), TGFα (*tgfa*), HB-EGF (*hbegf*) and betacellulin (*btc*) are exclusively or primarily expressed in the oocyte whereas their common receptor, EGFR (*egfr*), is exclusively expressed in the follicle layer, strongly suggesting an EGF family-mediated paracrine regulation of the somatic follicle cells by the oocyte. On the contrary, activin subunits, βA (*inhbaa*) and βB (*inhbb*), are exclusively expressed in the follicle layer while their receptors, *acvr1b*, *acvr2a* and *acvr2b*, are predominantly expressed in the oocyte, suggesting an activin-mediated follicle cell-to-oocyte signaling in the zebrafish follicle [31]. The present study provided evidence for a potential Kit-mediated paracrine mechanism in the zebrafish follicle, which may mediate both follicle cell-to-oocyte and oocyte-to-follicle cell signaling.

Similar to the situation in mammalian models [8], [9], Kitlga was expressed only in the follicle layer and Kita only in the oocyte, suggesting a Kitlga/Kita-mediated paracrine signaling from the somatic follicle cells to the oocyte. This idea was further supported by the experiment on receptor specificity. Recombinant Kitlga preferentially activated Kita but not Kitb. Different from the Kit system in mammals, there exist two forms of Kit ligand (Kitlga and Kitlgb) and receptor (Kita and Kitb) in teleosts [23]. In contrast to Kit ligand/Kit in mammals and Kitlga/Kita in the zebrafish, Kitlgb was found to exist in the oocyte and Kitb in the follicle layer, pointing to the possibility for a unique Kitlgb/Kitb paracrine pathway in the zebrafish follicle that mediates the control of the follicle cells by the oocyte. Again, this idea was supported by the receptor specificity data that Kitlgb specifically activated Kitb but not Kita. Different from Kita, however, Kitb still had a weak response to Kitlga, which was demonstrated by both receptor activation assay in the COS cells and MAPK phosphorylation in cultured zebrafish follicle cells. This weak interaction between Kitlga and Kitb may be analogous to the Kit-mediated granulosa-theca cell interaction in mammals [23]. The receptor specificity demonstrated in the present study will be better confirmed in the future by using purified Kitlga and Kitlgb as the recombinant cell lines used in the present study had different production yields. In spite of this, the present study strongly supports the existence of a Kit-mediated bi-directional communication between the somatic follicle cells and the oocyte.

As demonstrated by synteny and phylogenetic analysis in our previous report [23], Kitlga/Kitlgb and Kita/Kitb were likely derived from the third round genome duplication specific to ray-finned fish [48], [49]. Unlike many duplicated genes which possess redundant functions, both forms of Kit (Kita and Kitb) and Kit ligand (Kitlga and Kitlgb) in the zebrafish showed distinct expression patterns during folliculogenesis, particularly in periovulatory stage, implying functional differentiation of the Kit system in the ovary. This was further supported by the present study on receptor specificity of the Kit system in the zebrafish and their spatial distribution within the follicle. The oocyte is likely subject to the regulation by Kitlga derived from the follicle cells via Kita on the oocyte, which is analogous to the KITL/KIT signaling established in mammalian ovaries [39]. However, in contrast to the mechanism in mammals, the additional pathway mediated by Kitlgb/Kitb may represent a unique paracrine mechanism that signals back to the follicle cells from the oocyte.

The exact roles of the two Kit-mediated pathways in the zebrafish follicle are largely unknown. Our recent study showed that the expression of *kitlga* showed a significant decline in mature follicles [23], suggesting a potential role in final oocyte maturation. On the other hand, the follicle cells may be subject to the regulation by Kitlgb from the oocyte. In the light of the specificity of Kitlgb for Kitb and the high levels of *kitb* expression in the follicle layer after oocyte maturation and *kitlgb* in the oocyte before spawning [23], we surmised that Kitlgb might be involved in triggering important events in follicle cells in this period.

It has been reported that the binding of Kit ligand to Kit can activate MAPK pathway in mammals [50]–[53]. Using MAPK as a responsive marker, we monitored the change of MAPK phosphorylation in both cultured follicle cells and ovulated oocytes in response to recombinant zebrafish Kit ligands, which would not only confirm the biological activity of the CHO cell-derived recombinant proteins in the zebrafish but also serve to verify the distribution of the two Kit receptors in the follicle. Both Kitlga and Kitlgb significantly induced MAPK phosphorylation in cultured follicle cells, but Kitlga was much less potent, which was in agreement with the lack of Kita receptor in the follicle layer. Its low activity on MAPK was likely due to its weak activation of Kitb receptor expressed in the follicle cells as demonstrated by the receptor assay.

In contrast to the expression of Kitb in the follicle cells, Kita was expressed in the oocytes and it could only be activated by Kitlga. To confirm this finding, we used ovulated and dechorionated eggs as the material to examine MAPK response to Kit ligands. During ovulation, the oocytes are freed from the follicle layers, making it possible to study oocyte response without interference from the follicle cells. To ensure quick access to the oocytes during short-time treatment by drugs, we removed the chorion before treatment. The functionality and responsiveness of the denuded eggs were confirmed by IGF-I and EGF-induced MAPK phosphorylation. In agreement with our previous evidence for IGF-IR expression in both oocytes and follicle layer (data not shown), IGF-I significantly increased the phosphorylation level of MAPK both in the full-grown follicles and the ovulated oocytes. Similarly, the potent effect of EGF on MAPK phosphorylation in intact follicles also agreed well with the existence of its receptor EGFR in the follicle layer [31]. To our surprise, EGF also increased MAPK phosphorylation in the ovulated eggs free of follicle cells although its potency was lower than that of IGF-I. This could be due to the existence of other members of EGFR family in the oocytes [31]. Also, we cannot rule out the possibility that EGFR may be expressed in mature ovulated eggs as our previous work on EGFR distribution was carried out in immature non-ovulated follicles [31]. Furthermore, a recent study also provided evidence for the existence of EGFR protein in the zebrafish oocytes [54]. These lines of evidence also indicate that the ovulated oocytes can be used for testing direct effects of regulatory factors on oocytes. Different from the activation of MAPK by both Kitlga and Kitlgb through Kitb in the follicle cells, the phosphorylation level of MAPK in the ovulated oocytes was only increased by Kitlga, which agreed well with the observation that the oocyte expressed Kitlga-specific receptor Kita, but not Kitb. The activation of MAPK by Kitlga and Kitlgb in zebrafish follicle cells and ovulated oocytes not only confirmed the receptor specificity demonstrated in the present study but also provided supportive evidence for the bi-directional paracrine pathways mediated by the Kit system in the zebrafish follicle.

After demonstrating receptor specificity of the zebrafish Kit system, we performed a structural modeling analysis with the aim to elucidate the mechanism that determines the receptor specificity. As reported in previous studies [7], [45], mouse KIT is negatively charged at site I and positively charged at site II and III, whereas KITL has an opposite pattern of charge distribution, demonstrating a strong charge complementarity between the receptor and ligand at these sites. Charge complementarity, together with abundant hydrogen bonds between polar amino acids and interaction between hydrophobic amino acids at three sites, is believed to be responsible for the recognition and binding of KITL and KIT [7], [43]–[45]. Compared with the charge usage in mouse KITL/KIT interaction, the situation in the zebrafish appeared more complicated. Due to the existence of equal negative and positive charges, zebrafish Kita and Kitb seemed to be neutral at site II and I respectively, which did not contribute to their interaction with the ligands as mouse KITL does. Most important of all, the site I of Kita uses positive charge and site II of Kitb is rich in negative charge, which would exclude site I or site II of mouse KITL with the same charge. The loss of interaction at these two sites is likely responsible for the lack of activity of mouse KITL on zebrafish Kita and Kitb. On the other hand, Kitlga was rich in negative charge at both site I and III, while its preferred receptor Kita was reversely charged at these sites. The site II of Kita is neutral, but like that between mouse KITL and KIT [43], [44], the abundance of hydrophobic amino acids and hydrogen bonds at this site between Kitlga and Kita provided an alternative mechanism for interaction in the absence of charges. If acting on Kitb, Kitlga would have weak electrostatic interaction at site II and III, explaining its weak activation of Kitb. In comparison with Kitlga, Kitlgb shows strong charge complementarity with Kitb at site II and III, but only weak complementarity at these sites with Kita. This would explain why Kitlgb activated Kitb but had no activity on Kita. It is interesting to note that Kitb may not be able to interact with either Kitlga or Kitlgb at site I due to its lack of net changes at this site. As discussed above, although similar situation exists between Kitlga and Kita at site II, the lack of electrostatic interaction at this site seems to be compensated by hydrophobic interaction and hydrogen bonds. However, there is limited hydrogen bond formation and hydrophobic interaction between Kitlgb and Kitb at site I due to the limited number of amino acids at this site as compared to site II and III, which is the same as the situation in mammals [43], [44]. Therefore the binding affinity between Kitlgb and Kitb at site I is expected to be weak. This may account for the result that Kitlgb was less potent in stimulating its receptor Kitb as compared to Kitlga-induced phosphorylation of Kita. The structural analysis of zebrafish Kit system provides a theoretical explanation for the receptor specificity and binding potency observed in the present study. Further experimental approaches such as mutagenesis will be valuable to verify the ideas derived from the molecular modeling.

Previous studies have shown that the two pairs of zebrafish Kit ligands and receptors are derived from genome duplication specific to ray-finned fish [23]–[25]. Unlike many duplicated genes with similar expression pattern or redundant function, members of the zebrafish Kit system demonstrated specific expression profiles during folliculogenesis and in particular at periovulatory period [23], suggesting different functions for both ligands or receptors. In the present study, we further characterized their spatial distribution within the follicle and determined their receptor binding specificity. All these lines of evidence suggest the existence of a Kit-mediated bi-directional communication system in the follicle, i.e., Kitlga-Kita and Kitlgb-Kitb. Further evidence for the functional divergence of Kitlga and Kitlgb in the follicle came from our assay on oocyte maturation. Similar to the inhibitory effect of KITL on GVBD in the mouse [14], [15], [55], [56], both Kitlga and Kitlgb suppressed spontaneous GVBD in vitro, indicating that the two pathways had conserved function in controlling final oocyte maturation although the two Kit ligands work at different sites in the follicle. The function of Kitlga-Kita within the follicle is analogous to that of KITL-KIT in mammals because of their similar distribution in the follicle. However, the observation that the oocyte-derived zebrafish Kitlgb also had similar effect is interesting as the effect is likely exerted through Kitb located on the follicle cells. The involvement of Kitlgb-Kitb in regulating oocyte maturation is also supported by our previous report that *kitb* mRNA level in the zebrafish follicle surged at the full-grown stage before final maturation [23]. In contrast to the lack of effect on DHP-induced oocyte maturation by Kitlga, Kitlgb significantly promoted DHP-induced GVBD. This further suggests a neofunctionalization of the Kit ligand homologues in teleosts during evolution. Our speculation is that, unlike the Kitlga-Kita pathway that blocked oocyte maturation, the Kitlgb-Kitb pathway might increase the maturational competence of oocytes while suppressing their spontaneous maturation, which would promote oocyte maturation once DHP is secreted from the follicle layer. Considering that the effect of Kitlgb-Kitb on oocytes may be indirect, we believe that some factors may be activated by this pathway in the follicle cells, which in turn act on the oocytes to signal for the suppression of basal maturation but promotion of DHP-induced maturation. Our previous studies have shown that activin has potent effect on the acquisition of oocyte maturational competence [57] and activin subunits are exclusively expressed in the follicle cells whereas its receptors abundantly expressed in the oocyte [31], [58]. This makes activin a potential molecule in the follicle cells that mediate the actions of other regulatory factors. Whether Kitlgb exerts its action on maturational competence via activin in the follicle cells will be an interesting issue to investigate in the future. Although the action mechanisms and physiological relevance of the two Kit signaling pathways in the zebrafish follicle are unclear, our results showed that Kitlga-Kita and Kitlgb-Kitb are functionally divergent in the regulation of oocyte maturation.

**Figure 14:**
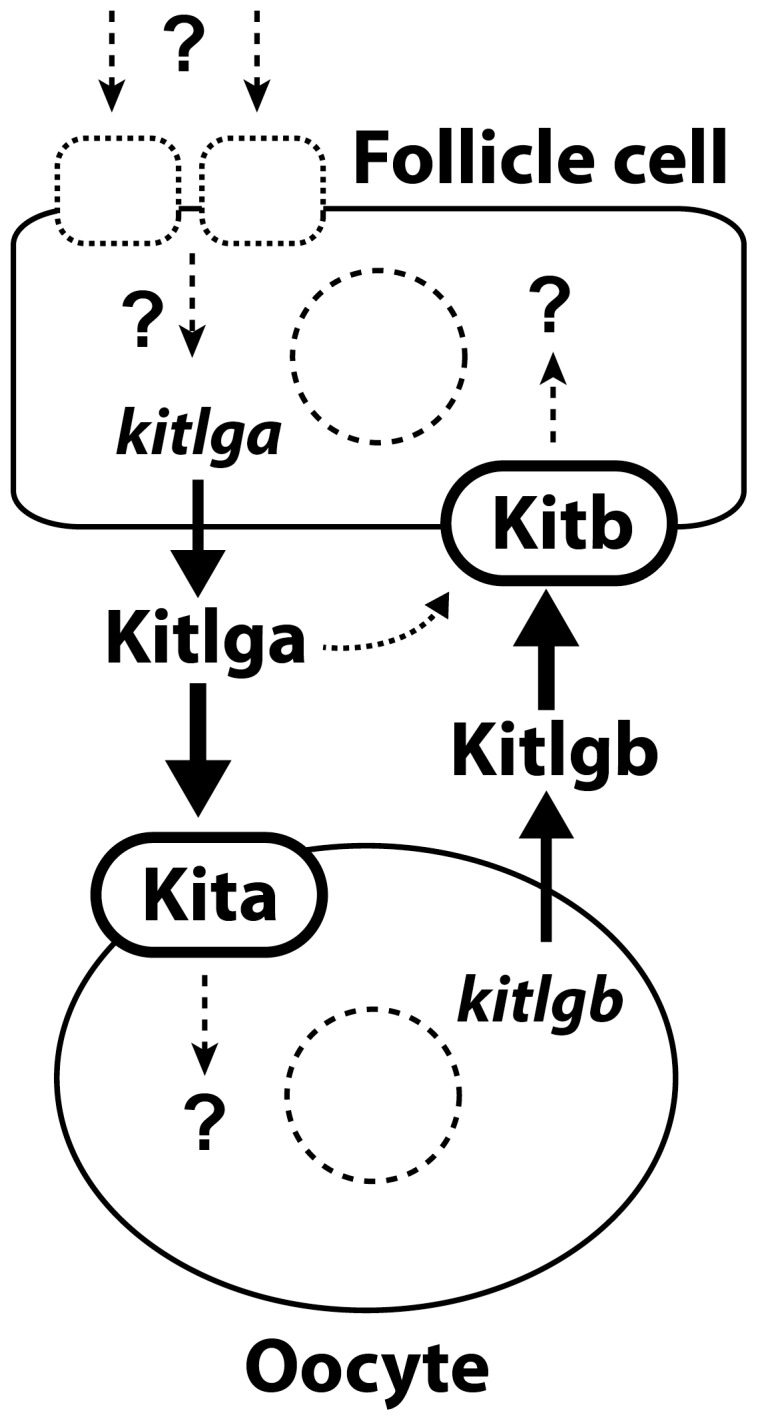
The hypothetical model for the existence of a Kit-mediated bi-directional communication network in the zebrafish ovarian follicle. The follicle cell-derived Kitlga specifically targets its receptor Kita on the oocyte with a minor action on Kitb on the follicle cells as well, similar to the KITL-KIT system in mammals. The Kitlgb, however, is exclusively expressed in the oocyte and it acts on its specific receptor Kitb on the follicle cells, representing a potential paracrine signaling pathway for the oocyte to control the follicle cells. The expression of Kitlga in the follicle cells is likely subject to regulation by various endocrine and/or paracrine factors, therefore mediating the actions of these factors on the oocyte.

In summary, the present study demonstrated the spatial distribution of all Kit system members in the zebrafish ovary. The *kitlga* and *kitb* are exclusively expressed in the follicle layer, while *kitlgb* and *kita* only in the oocyte. Using CHO cells as the bioreactor, we produced recombinant zebrafish Kitlga and Kitlgb. Analyses in the COS-1 cells, zebrafish primary follicle cells, and ovulated oocytes confirmed their biological activity and receptor specificity. Two opposite paracrine pathways involving the Kit system in the zebrafish ovary were suggested. Kitlga from the follicle cells preferably activated Kita in the oocyte and weakly activated Kitb on the follicle cells. Kitlgb from the oocyte, however, exclusively activated Kitb in the follicle cells without any effects on Kita (Fig. 14). The molecular mechanism for the receptor specificity was further explored by three-dimensional protein structure analysis and molecular modeling. Finally, we showed differential functions of Kitlga and Kitlgb in controlling oocyte maturation. This study provides lines of evidence, including localization, receptor specificity, signal transduction and function, for the existence of a Kit-mediated bi-directional communication system in the zebrafish follicle.
